# Collateral Formation from Left Lateral Thoracic Artery to the Adamkiewicz Artery

**DOI:** 10.1055/s-0040-1721748

**Published:** 2021-03-24

**Authors:** Toshihiro Fukui, Jun Takaki, Ken Okamoto

**Affiliations:** 1 Department of Cardiovascular Surgery, Kumamoto University Hospital, Kumamoto, Japan

**Keywords:** Adamkiewicz artery, collateral, thoracoabdominal aortic aneurysm

## Abstract

A 68-year-old man who had undergone descending thoracic aortic replacement was referred to our hospital with a thoracoabdominal aortic aneurysm. During the original surgery, the Adamkiewicz artery was directly reconstructed. However, multidetector row computed tomography showed occlusion of the reconstructed artery at its orifice, with supply by a collateral vessel from the left lateral thoracic artery. With careful incision to avoid damage to the collateral vessel, no postoperative neurological deficit was observed.

## Introduction

Spinal cord ischemia (SCI) is a critical complication of a postoperative thoracoabdominal aortic aneurysm (TAAA). The Adamkiewicz artery (AKA) is the dominant feeder of the spinal cord, and numerous surgical reports have stressed the importance of preserving the AKA in patients undergoing TAAA repair. 1 Here, we report a patient undergoing TAAA repair who had a collateral from the left lateral thoracic artery to the AKA that was occluded after prior descending thoracic artery replacement.

## Case Presentation




**Video 1** Preoperative 3-D computed tomography showing a collateral vessel from the left thoracic artery to the Adamkiewicz artery.


A 68-year-old man with hypertension was admitted to our hospital with a TAAA. He had undergone a nephrectomy for left renal cell cancer 2 years prior and descending thoracic aortic repair for a ruptured Type-B acute aortic dissection at another hospital 8 years ago. At that operation, a reconstruction of the 11th intercostal artery, which was identified as the AKA, was performed. Follow-up computed tomography (CT) was performed annually, which showed gradual enlargement of the thoracoabdominal aorta. Because the maximum diameter of the aneurysm reached 62 mm, we planned to perform a TAAA repair.

**Fig. 1:**
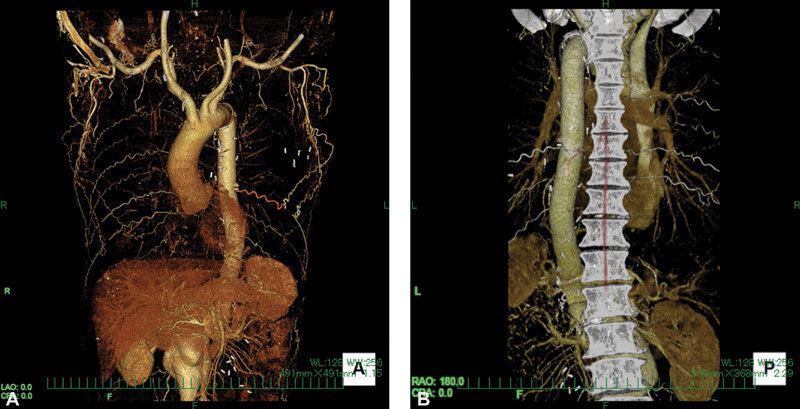
Preoperative computed tomography showing a collateral vessel from the left lateral thoracic artery to the Adamkiewicz artery. ( **A** ) Anterior view and ( **B** ) posterior view.

Preoperative CT findings demonstrating a replaced thoracic aorta from the distal aortic arch to just above the celiac artery are shown in Fig. 1 and Video 1 . Unexpectedly, the reconstructed intercostal artery was occluded. However, the AKA was supplied by a collateral vessel from the left lateral thoracic artery that was clearly visualized by CT. Because the collateral vessel was located in the left lateral thoracic wall, we planned a careful approach to the aorta to avoid injury to this vessel during the incision. A catheter for cerebrospinal fluid drainage was inserted into the lumbar region a day before the surgery.

During the operation, a left-edge skin incision was made 3-cm apart from the inferior angle of the scapula. The chest was then opened through the left eighth intercostal space. We carefully dissected the left lung and the prosthetic graft, as they were severely adhered to the surrounding tissue. Cardiopulmonary bypass was initiated through the left femoral vein to the left femoral artery. A segmental-staged aortic clamp was applied under a partial cardiopulmonary bypass.

The aorta was clamped at the prosthetic aortic graft proximal to the previous distal anastomosis and at the native aorta just above the celiac artery. The previous distal anastomosis was transected, and a 20-mm Dacron graft with 8-mm side branches was anastomosed to the previous graft with a 4–0 polypropylene suture. The native aortic clamp was moved to the terminal aorta, and the aorta was incised. The celiac and superior mesenteric arteries were perfused using 8-Fr size balloon tipped catheters via a single roller pump. A cold ringer solution was infused into the bilateral renal artery. Each visceral artery was individually transected and anastomosed to the side branches of the prosthetic graft using a 5–0 polypropylene suture. After reconstruction of the visceral arteries, the prosthetic aortic clamp was moved to the distal end of the prosthetic graft to perfuse the side branches. The right external iliac artery and left common iliac artery were reconstructed with a 16- to 8-mm **Y** -shaped Dacron graft, and the right internal iliac artery was reconstructed using another 8-mm Dacron graft. No intercostal or lumber arteries were reconstructed. The operation time was 305 minutes.

**Fig. 2:**
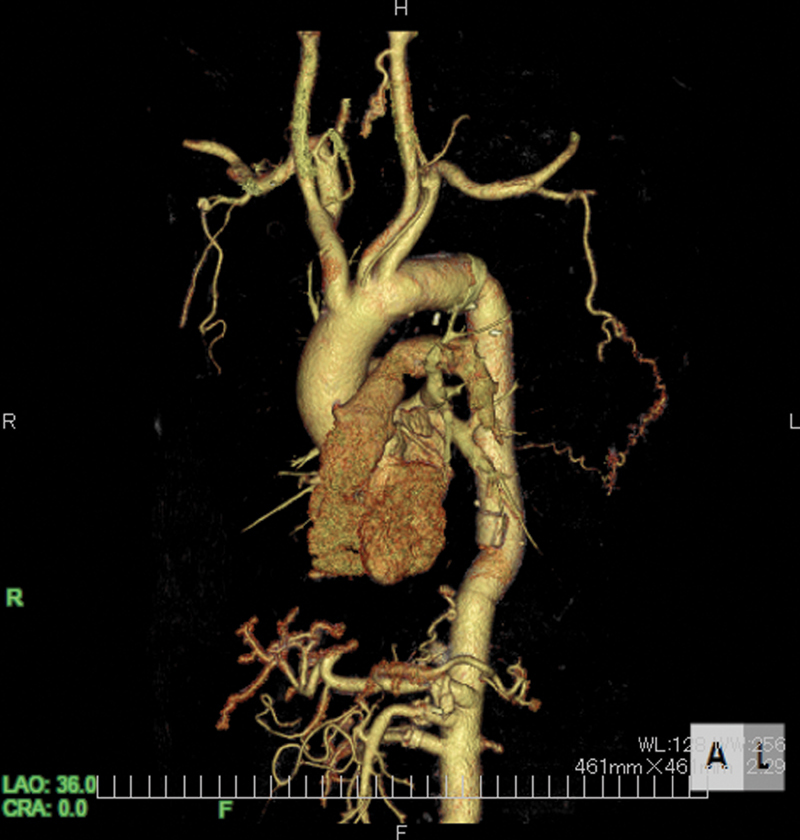
Postoperative computed tomography showing an intact collateral vessel from the left lateral thoracic artery to the Adam-Kiewicz artery.

After the operation, the patient had no neurological deficits including paraplegia. Postoperative CT demonstrated the preserved collateral vessel from the left lateral thoracic artery to the AKA ( Fig. 2 ). He was discharged from hospital for rehabilitation on the 18th postoperative day.

## Discussion

SCI is a devastating complication after thoracoabdominal aortic repair. Despite development of numerous strategies to minimize the risk of SCI, the incidence of SCI is 3 to 10%. 2 A possible cause of SCI during surgery is failure to reestablish the blood supply to the spinal cord, and the importance of reconstruction of the intercostal or lumbar arteries related to the AKA is widely reported. 3 In the present case, preoperative three-dimensional CT revealed that the left lateral thoracic artery was a feeding artery to the AKA as a collateral artery. The left lateral thoracic artery is a rare collateral source of the AKA. Yoshioka et al 4 evaluated the collateral pathways to the AKA using multidetector row CT and found that from 75 collateral pathways there were 11 cases with collateral pathways located in the thoracic wall, including seven in the thoracodorsal artery, three in the internal thoracic artery, and one in the inferior epigastric artery, while the lateral thoracic artery was not detected as a collateral artery to AKA.

Detection rates for AKA are as high as 80 to 90% using 64-detector row CT. 4 Further, using a 320-detector row CT, the collateral circulation was identified in 83% of patients with occlusion of the segmental artery from which the AKA originated. 4 We believe that multidetector row CT should be routinely performed, particularly in patients with a previous thoracic or abdominal aortic operation, because of the risk of development or change of a collateral artery of the AKA. In our case, despite reconstruction of the 11th intercostal artery during a prior surgery, its orifice was occluded before the patients' recent operation, and the left lateral thoracic artery was unexpectedly detected as a collateral. If we had performed a conventional approach with a posterolateral thoracotomy without multidetector row CT, the incision may have damaged the left lateral thoracic artery. Thus, we took particular care when making the skin incision to avoid damage to the collateral vessel.
